# Rotational method simplifies 3-dimensional measurement of left atrial appendage dimensions during transesophageal echocardiography

**DOI:** 10.1186/s12947-016-0079-y

**Published:** 2016-08-24

**Authors:** Chaim Yosefy, Yulia Azhibekov, Boris Brodkin, Vladimir Khalameizer, Amos Katz, Avishag Laish-Farkash

**Affiliations:** 1 Department of Cardiology, Barzilai Medical Center, Ben-Gurion University of the Negev, Ashkelon, Israel; 2 Department of Imaging, Barzilai Medical Center, Ben-Gurion University of the Negev, Ashkelon, Israel; 3 Noninvasive Cardiology Unit, Barzilai Medical Center, Ashkelon, 78306 Israel

**Keywords:** 3-dimensional transesophageal echocardiography, Left atrial appendage, Imaging, Computed tomography, Rotation

## Abstract

**Background:**

Not all echo laboratories have the capability of measuring direct online 3D images, but do have the capability of turning 3D images into 2D ones “online” for bedside measurements. Thus, we hypothesized that a simple and rapid rotation of the sagittal view (green box, x-plane) that shows all needed left atrial appendage (LAA) number of lobes, orifice area, maximal and minimal diameters and depth parameters on the 3D transesophageal echocardiography (3DTEE) image and LAA measurements after turning the images into 2D (Rotational 3DTEE/“Yosefy Rotation”) is as accurate as the direct measurement on real-time-3D image (RT3DTEE).

**Methods:**

We prospectively studied 41 consecutive patients who underwent a routine TEE exam, using QLAB 10 Application on EPIQ7 and IE33 3D-Echo machine (BORTHEL Phillips) between 01/2013 and 12/2015. All patients underwent 64-slice CT before pulmonary vein isolation or for workup of pulmonary embolism. LAA measurements were compared between RT3DTEE and Rotational 3DTEE versus CT.

**Results:**

Rotational 3DTEE measurements of LAA were not statistically different from RT3DTEE and from CT regarding: number of lobes (1.6 ± 0.7, 1.6 ± 0.6, and 1.4 ± 0.6, respectively, *p* = NS for all); internal area of orifice (3.1 ± 0.6, 3.0 ± 0.7, and 3.3 ± 1.5 cm^2^, respectively, *p* = NS for all); maximal LAA diameter (24.8 ± 4.5, 24.6 ± 5.0, and 24.9 ± 5.8 mm, respectively, *p* = NS for all); minimal LAA diameter (16.4 ± 3.4, 16.7 ± 3.3, and 17.0 ± 4.4 mm, respectively, *p* = NS for all), and LAA depth (20.0 ± 2.1, 19.8 ± 2.2, and 21.7 ± 6.9 mm, respectively, *p* = NS for all).

**Conclusion:**

Rotational 3DTEE method for assessing LAA is a simple, rapid and feasible method that has accuracy similar to that of RT3DTEE and CT. Thus, rotational 3DTEE (“Yosefy rotation”) may facilitate LAA closure procedure by choosing the appropriate device size.

## Background

Ninety percent of clots in patients with non-valvular atrial fibrillation (AF) occur in the left atrial appendage (LAA). The shape and location of LAA allow for stasis of blood in AF, mitral stenosis (MS), and other low cardiac output conditions. Clots may remain hidden because of the three-dimensional (3D) complexity of the LAA [1, 2]. Complex LAA morphology characterized by an increased number of LAA lobes (≥3) was associated with the presence of LAA thrombus independently of clinical risk and blood stasis [1].

Over the last years, minimally invasive epicardial techniques and catheter-based transseptal techniques have been developed for occlusion of the LAA orifice to reduce stroke risk [3–5]. These devices and procedures may provide an alternative to oral anticoagulation (OAC) for AF patients at high risk for stroke but with contraindications for chronic OAC [6–8].

Accurate knowledge of LAA anatomy and dimensions has become a key guiding stage before introducing LAA closure devices [7, 9, 10]. LAA assessment should be done prior to procedure [7, 11–13]. Currently, 2D transesophageal echocardiography (2DTEE) at a cut plane angulation of 135° is the recommended method to size maximal LAA orifice diameter before introducing a percutaneous LAA closure device [9, 10, 14, 15]. However, 2DTEE does not adequately allow complete spatial visualization of the LAA [11–13, 16]. Thus, three-dimensional imaging modalities [11–13] such as cardiac magnetic resonance imaging (MRI), computed tomography (CT), and Real-Time-3-Dimensional Transesophageal Echocardiography (RT3DTEE) may be more accurate [15, 17–19].

Recent trials show better performance of RT3DTEE for the assessment of LAA anatomy compared with 2DTEE regarding LAA orifice area, LAA ejection fraction calculation, and LAA volume [15, 20–22]. Our group showed that bedside direct online RT3DTEE measurements of LAA maximal orifice diameter are more accurate than 2DTEE measurements and are as accurate as CT as gold standard [16]. Thus, direct RT3DTEE measurements may facilitate LAA closure procedure by choosing the appropriate device size. However, not all echo laboratories have the capability of using this method that directly measures the 3D images (RT3DTEE) but yet have the capability to online turn 3D images into 2D ones for bedside measurements.

We analyzed LAA measurements using a simpler and faster 3D method: after conversion of the 3D image into three 2D planes (X,Y,Z), the operator uses a 360° rotation of the sagittal plane (green box, x-plane), that enables him to rapidly choose the image which shows all the LAA parameters needed for the introduction of the invasive procedure in one single “stop shop” image (Rotational 3DTEE/“Yosefy Rotation”). Our aim was to validate the accuracy of Rotational 3DTEE versus the former direct online RT3DTEE method for LAA assessment.

## Methods

### Study population

A total of 41 consecutive patients who underwent a routine indicated 3DTEE and 64-slice CT, either before pulmonary vein isolation (PVI) ablation (*n* = 34) for precise definition of LA and pulmonary veins anatomy, or for workup of pulmonary embolism (PE) (*n* = 7) (Table 1). In this group of patients, 64-slice CT was used as reference technique to test the accuracy of 3DTEE-derived measurements of LAA parameters. We compared Rotational 3DTEE LAA measurements versus RT3DTEE.

**Table 1:** Baseline demographics and echocardiographic characteristics of the study population

	Study population (*n* = 41)
Age (years)	62.9 ± 12.7
Male	21 (51 %)
BSA (m^2^)	1.9 ± 0.2
Height (meters)	1.7 ± 0.1
Weight (kg)	84.1 ± 15.9
Indication of CT	Before PVI – 34
	PE workup – 7
Echocardiographic measurements (RT3DTEE)	
LVEF (est. %)	59.9 ± 6.9
LPW-D (mm)	10.1 ± 0.2
IVS-D (mm)	10.8 ± 2.4
LVESD (mm)	31.3 ± 3.7
LVEDD (mm)	48.8 ± 4.5
Pulmonary pressure (mmHg)	33.0 ± 14.0
RA area (cm^2^)	17.6 ± 4.5
LA area (cm^2^)	22.7 ± 5.3
LA diameter (ap) (mm)	39.8 ± 6.8
Ascending aorta diameter (mm)	32.4 ± 3.4
Aortic root diameter (mm)	30.4 ± 3.2

*BSA* body surface area, *CT* computed tomography, *est* estimated, *IVS-D* inter-ventricular septum diameter, *LPW-D* left posterior ventricular wall diameter, *LVEDD* left ventricular end diastolic diameter, *LVEF* left ventricular ejection fraction, *LVESD* left ventricular end systolic diameter, *PE* pulmonary embolism, *PVI* pulmonary vein isolation, *RT3DTEE* real-time 3-dimensional transesophageal echocardiography

### Echocardiography

Forty-one consecutive patients (out of 43 patients) who underwent the routine echocardiography exam using EPIQ7 and iE33 echo machine (Philips Medical Systems, Andover, MA) between January 2013 and December 2015 and had a good echogenic window, were included in the study. All images were digitally stored for offline analysis (QLAB 10.0 cardiac 3DQ, Philips Medical Systems).

Internal area of LAA orifice, LAA depth, maximal LAA diameter, minimal LAA diameter, and number of LAA lobes were compared between Rotational 3DTEE and RT3DTEE and to the CT as the gold standard method. All echocardiographic data have been reviewed by a single operator (CY) who was blinded to CT results done by a single operator (YA).

### Transesophageal echocardiography

Transesophageal echocardiography (TEE) was performed using a commercially available fully sampled matrix-array TEE transducer and ultrasound system (X7–2 t Live 3D TEE transducer).

### Real-Time-3-Dimensional Transesophageal Echocardiography (RT3DTEE)

RT3DTEE imaging was performed acquiring the usual pyramidal data set large enough to include the entire LAA. The zoom mode was used to improve visualization of LAA.

The internal area of the LAA orifice, as well as the minimal and maximal diameters of the LAA orifice (D_max_ and D_min_, respectively), were measured directly from the original 3D views, along a plane connecting the origin of the left Circumflex artery to the roof of the LAA, below the ligament of Marshall, as previously shown by our group [16]. These measurements were assessed online using the EPIQ 7 echo machine (Philips Medical Systems, Andover, MA), since they could not be measured off-line on QLAB 10.0 software. The LAA depth (i.e., the longest distance from LAA orifice at the Circumflex artery level to the tip of the LAA) was measured off-line from the long-axes views, using dedicated software (QLAB 10.0). On these datasets we tried to measure LAA 3D volume using the same tracking method that we used for LA volume measurement, as previously described [23].

### Rotational 3DTEE

**Fig. 1:**
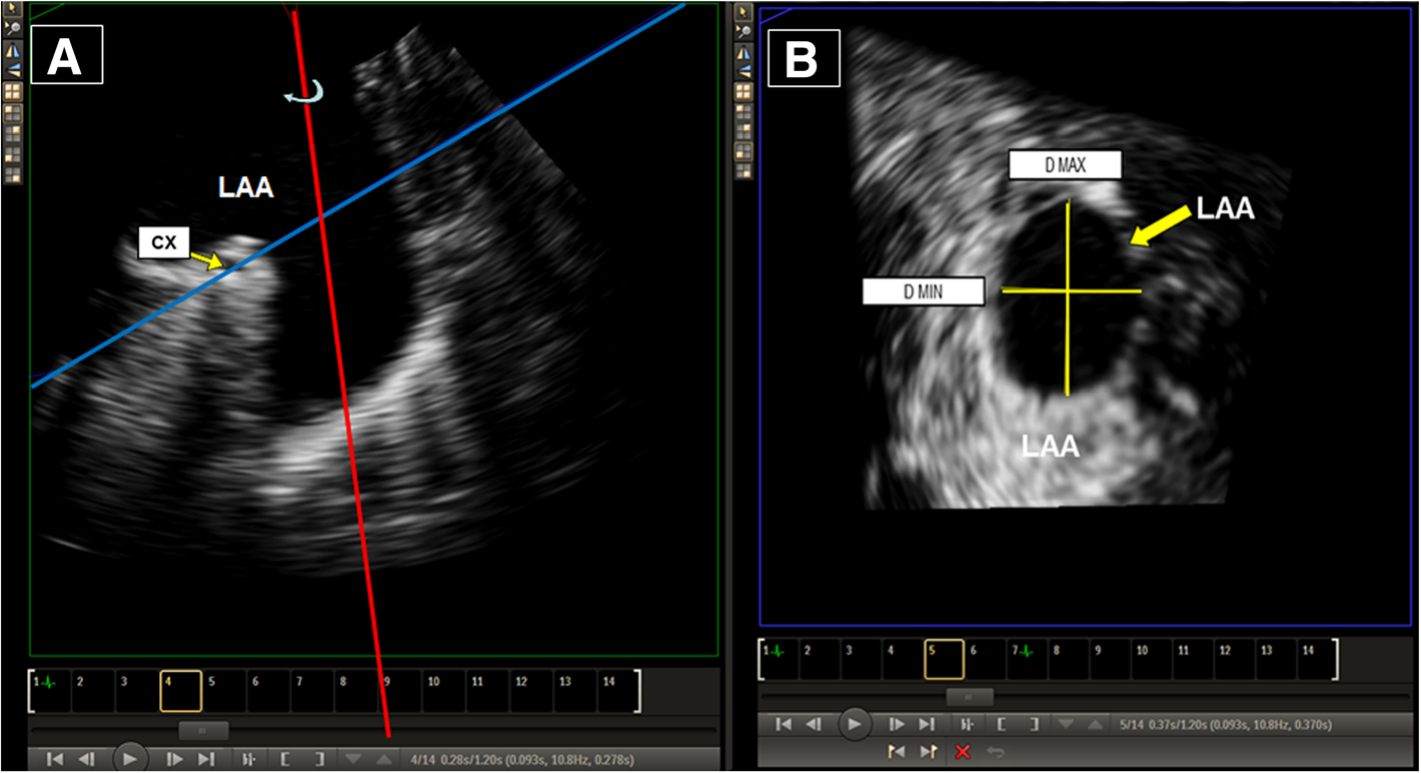
Three dimensional transesophageal echocardiography (3DTEE) measurements of left atrial appendage (LAA) using 360°, Rotational 3DTEE method (see text and panel **a**); and measurement of maximal (Dmax) and minimal (Dmin) diameters at the level of the circumflex (Cx) artery (panel **b** Video clip is attached)

Our 3D protocol for LAA dimensions measurements was as follows: Data acquisition included all the LAA in 3D zoom mode. We chose the 3DQ (and not 3DQadv) application and found the ECG guided end systole (i.e., end of T-wave) for the maximal LAA dimensions. We magnified multiplanar reconstruction (MPR) 2D images, then adjusted and cropped the lines to the optimal alignment. After optimizing the blue line to the circumflex artery level and decreasing the gain in the volume mode, we took the sagittal plane (green box, x-plane) and screened a 360° rotation. This 360° rotation (“Yosefy rotation”), is simpler and faster 3D method: it uses a rotation of the sagittal plane (in the green box, x-plane) that enables the operator to rapidly choose the image which gives him all the LAA parameters needed to introduce the invasive procedure in one single “stop shop” image (including: number of lobes - during the rotation; orifice area; maximal and minimal diameters - in the axial blue box; and depth - in the green box). In contrast to the usual time consuming MPR methods, in which each of the above parameters is taken at different angles (0°, 45°, 90°, 135°) and frames, using our rotation, we could easily find the “stop shop” image point where all the above LAA parameters are measured (Fig. 1).

All 3D measurements of LAA were performed at ventricular end-systole. All the patients included in our study were in sinus rhythm during the echocardiographic studies. Nevertheless, in patients who are in atrial fibrillation at the time of transesophageal echocardiography (TEE), we usually average measurements from ≥3 cardiac cycles.

### Sixty-four-slice computed tomography

**Fig. 2:**
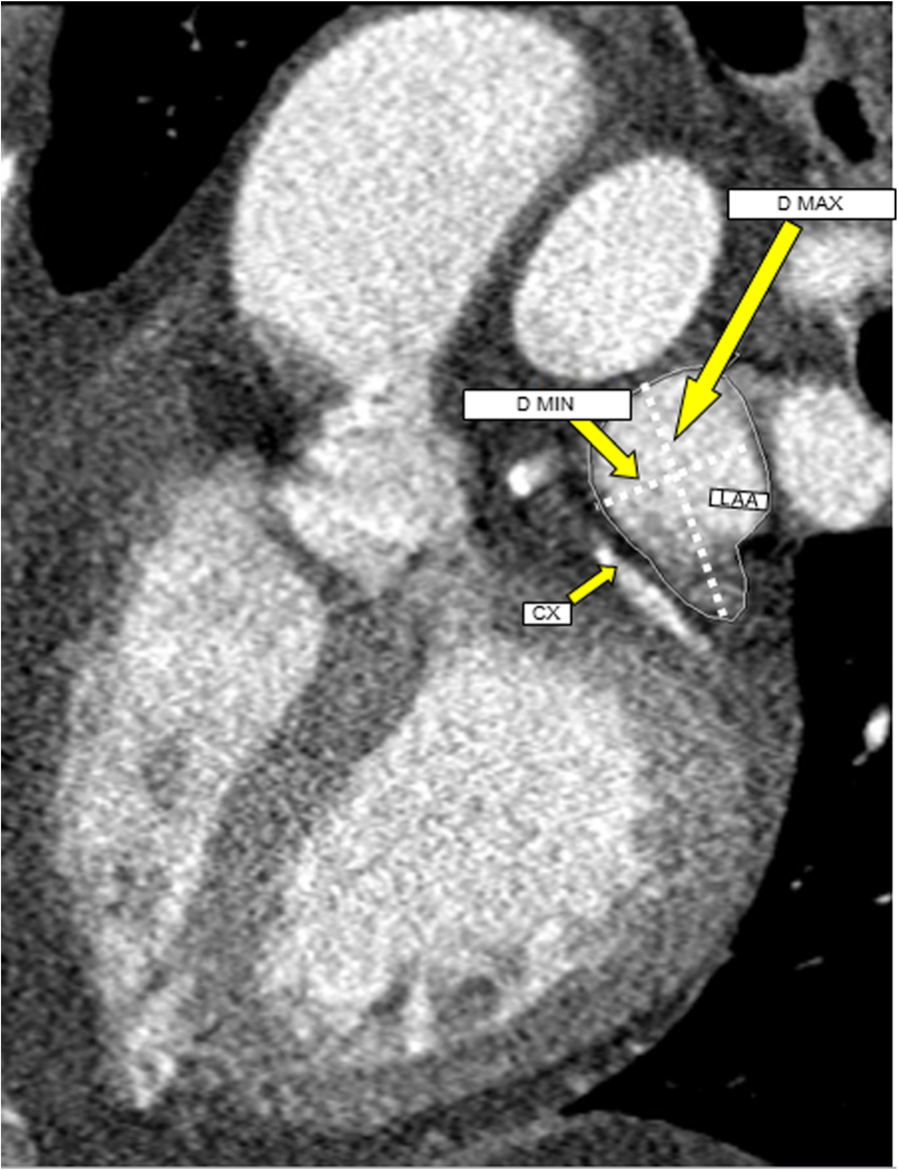
CT images of LAA orifice maximal diameter and area at the level of circumflex (Cx) artery (arrow). (D_max_ = largest diameter, D_min_ = minimal diameter)

All forty-one patients underwent clinically indicated 64-slice CT (Philips Brilliance CT 64 Power-Philips Medical Systems, Eindhoven, The Netherlands) within one week of transthoracic and transesophageal echocardiography. All patients were in sinus rhythm during the CT scan. A retrospective ECG-gating protocol was used. Scanning parameters were the following: detector collimation of 0.625 mm, total z-axis coverage of 40 mm per rotation, gantry rotation speed of 0.35 s, tube voltage of 120 kV, pitch of 0.16 to 0.24, and ECG modulated tube current ranging from 400 to 800 mA. The bolus tracking technique was used to trigger the acquisition, with a four-cavity view as the region of interest. A total of 70–100 mL of iodinated, nonionic contrast agent (Iomeron 350, Bracco Imaging S.p.A.) was injected continuously into the antecubital vein (100–120 mL at 5.0 mL/s). Scanning was initiated during a single breath hold for an acquisition time of 5 to 7 s. All images were reconstructed with an effective slice thickness of 0.625 mm. ECG-gating protocol reconstruction of the image data was performed starting from early systole (10 % of R-R interval) and ending at end-diastole (90 % of R-R interval) using 10 % steps. Reconstructed image data were transferred to a remote workstation (IntelliSpace Portal, Philips) for post-processing. For the purpose of the current study, image data sets reconstructed at end-systole (40 % of R-R interval) were used for analysis. Using MPR, measurements of the area of the LAA orifice were performed from the short-axis view as well as the maximum (D_max_) and minimum (D_min_) diameters (Fig. 2), and the maximum depth of LAA was measured as the longest distance from LAA orifice to the tip of LAA. All the patients included in our study were in sinus rhythm during the imaging studies. Nevertheless, in patients who are in atrial fibrillation at the time of CT scanning, we usually average measurements from ≥3 cardiac cycles.

### Statistical analysis

Continuous variables are presented as percentages and means ± standard deviation. Categorical data are presented as absolute numbers and percentages. Continuous variables were compared using independent Student *t*-test; categorical variables were compared using chi-square test or Fisher’s exact test. A 2-sided *p*-value < .05 was considered to indicate statistical significance for all tests. Analyses were carried out using SPSS version 21.0 statistical package (SPSS IBM. Inc.).

The intra-observer reproducibility of Rotational 3DTEE method for the measurement of all LAA parameters was demonstrated by performing the measurements of Rotational 3DTEE and CT in 10 randomized patients from our study by the same operator (CY for the TEE and YA for the CT), to assure they were not significantly different by paired *t* test. The measurements of LAA orifice area, maximal and minimal diameters and depth were calculated to obtain their SD and test the mean value versus 0.

Inter-observer variability was assessed between two observers in 10 patients selected randomly from our study patients. The measurements using Rotational 3DTEE and CT methods were obtained independently by two expert operators for each modality (CY and Doodit Mimon for the TEE and YA and Victor Lapis for the CT) blinded to the results of each other. Inter-observer variability was defined as the SD of the differences between observers and expressed as a percentage of the means.

## Results

### Baseline characteristics

Baseline characteristics of the study population (*n* = 41) are shown in Table 1. All patients had full readable Rotational 3DTEE and RT3DTEE images. Thus, there was 100 % feasibility of LAA assessment.

### Comparison between Rotational 3DTEE and RT3DTEE

**Fig. 3:**
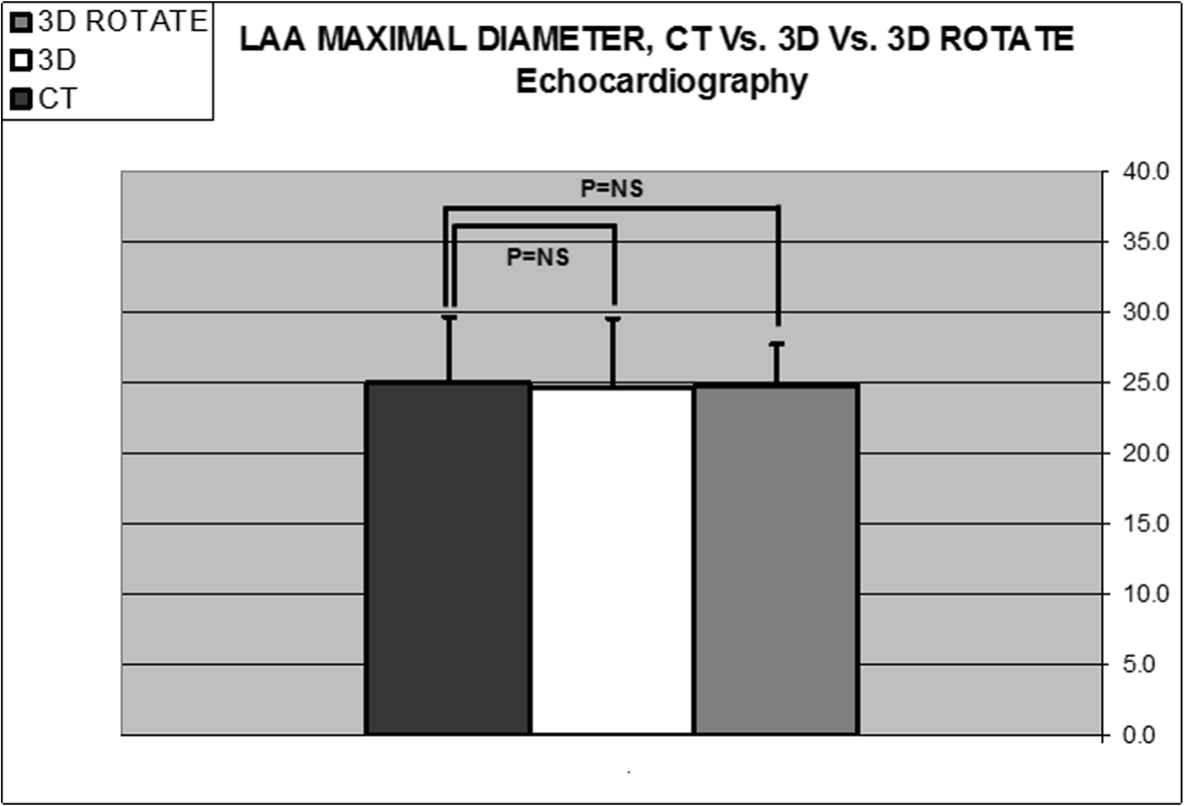
Histogram comparing LAA maximal diameter using different imaging methods (*n* = 41). Comparison between computed tomography (CT) (24.9 ± 5.8 mm), direct Real-Time 3-Dimensional Transesophageal Echocardiography (3D) (24.6 ± 5.0 mm), and Rotational 3DTEE (3D Rotate) (24.8 ± 4.5 mm), (*p* = NS for all)

**Fig. 4:**
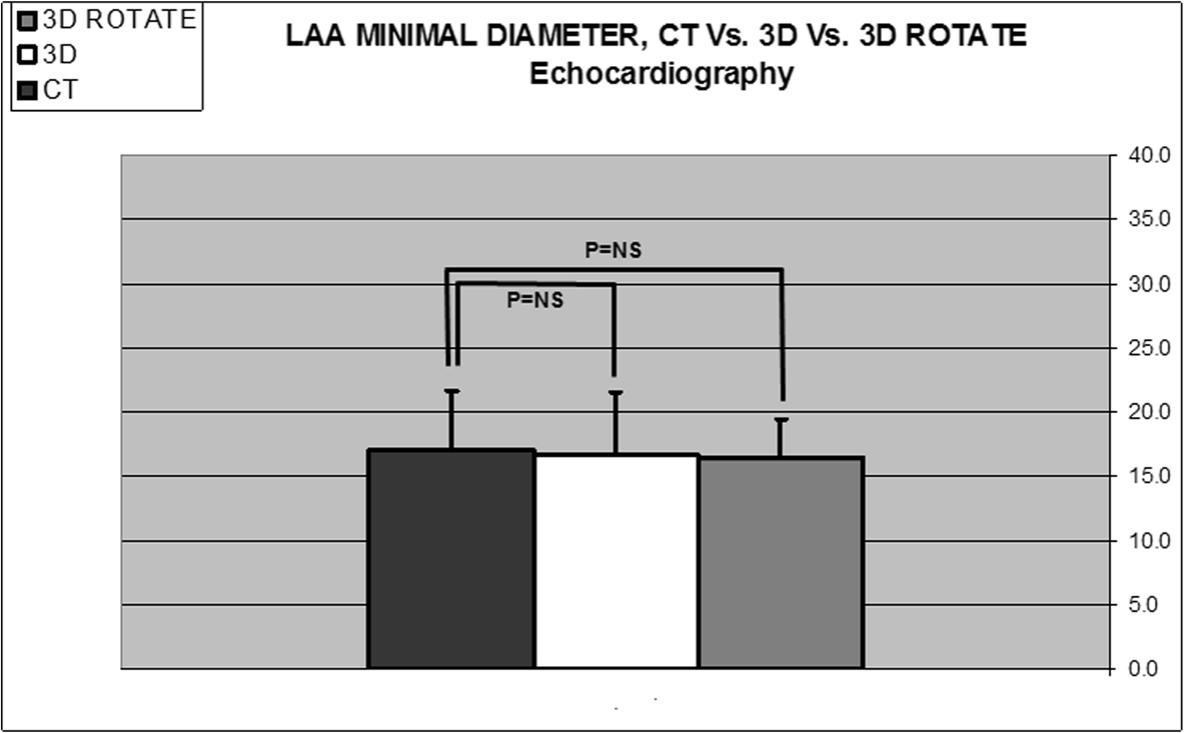
Histogram comparing LAA minimal diameter using different imaging methods (*n* = 41). Comparison between computed tomography (CT) (17.0 ± 4.4 mm), direct Real-Time 3-Dimensional Transesophageal Echocardiography (3D) (16.7 ± 3.3 mm), and Rotational 3DTEE (3D Rotate) (16.4 ± 3.4 mm), (*p* = NS for all)

**Fig. 5:**
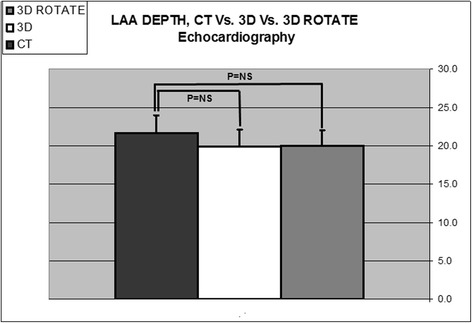
Histogram comparing LAA depth using different imaging methods (*n* = 41). Comparison between computed tomography (CT) (21.7 ± 6.9 mm), direct Real-Time 3-Dimensional Transesophageal Echocardiography (3D) (19.8 ± 2.2 mm), and Rotational 3DTEE (3D Rotate) (20.0 ± 2.1 mm), *(p* = NS for all)

Rotational 3DTEE measurements of LAA were not statistically different from RT3DTEE regarding: number of lobes (1.6 ± 0.7 and 1.6 ± 0.6, respectively, *p* = NS); area of orifice (3.1 ± 0.6 and 3.0 ± 0.7 cm^2^, respectively, *p* = NS); maximal LAA diameter (24.8 ± 4.5, 24.6 ± 5.0 mm, respectively, *p* = NS) (Fig. 3); minimal LAA diameter (16.4 ± 3.4, 16.7 ± 3.3 mm, respectively, *p* = NS) (Fig. 4); and LAA depth (20.0 ± 2.1 and 19.8 ± 2.2 mm, respectively, *p* = NS) (Fig. 5).

As in our previous study [16], LAA volume could not be measured directly using RT3DTEE due to inability of the technology to track the lobe borders.

### Comparison between Rotational 3DTEE and CT

Rotational 3DTEE measurements were not different from CT measurements regarding: number of LAA lobes (1.6 ± 0.7 and 1.4 ± 0.6, respectively, *p* = NS); area of orifice (3.1 ± 0.6 and 3.3 ± 1.5 cm^2^, respectively, *p* = NS); maximal LAA diameter (24.8 ± 4.5, 24.9 ± 5.8 mm, respectively, *p* = NS) (Fig. 3); minimal LAA diameter (16.4 ± 3.4, 17.0 ± 4.4 mm, respectively, *p* = NS) (Fig. 4); and LAA depth (20.0 ± 2.1 and 21.7 ± 6.9 mm, respectively, *p* = NS) (Fig. 5).

**Fig. 6:**
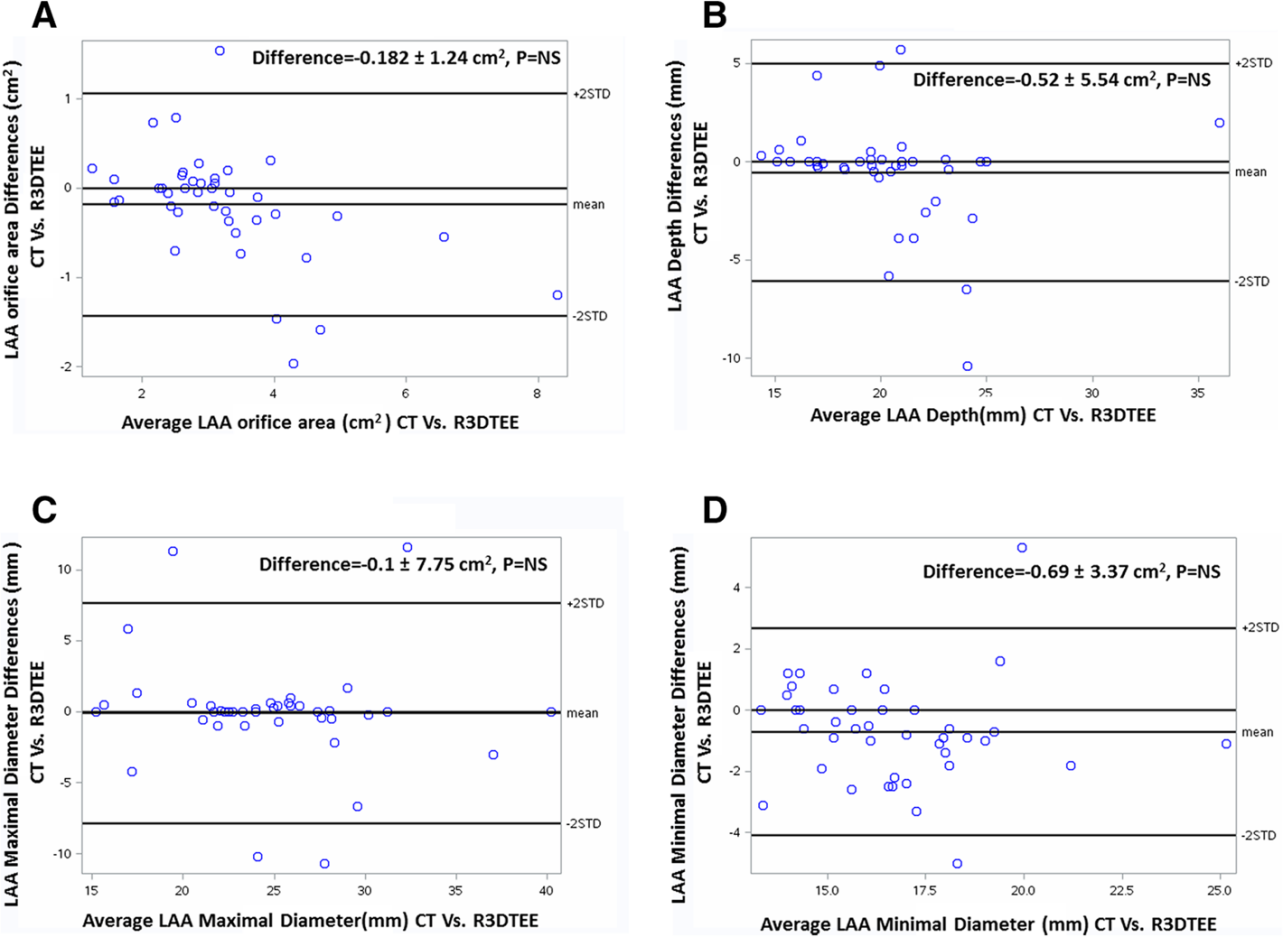
Bland-Altman analysis of differences between left atrial appendage (LAA) orifice area (**a**), depth (**b**), maximal (**c**), and minimal (**d**), diameters by CT scanning Vs. Rotational 3DTEE

The Bland-Altman analysis shows good correlation and low variability between LAA orifice area, maximal and minimal diameters and depth measured by Rotational 3DTEE and by CT scanning (Fig. 6a-d).

Inter-observer variability of LAA orifice area, maximal and minimal diameters and depth measured by Rotational 3DTEE and by CT scan methods was calculated and compared between the two observers. The variability of the mean values was 3.9, 4.1, 2.9 and 3.1 %, respectively, for the Rotational 3DTEE, and 3.2, 3.6, 2.7 and 3.8 %, respectively, for the CT. The observers concordance was with good agreement (*r* = 0.97, 0.95, 0.95 and 0.97, respectively) between the two methods.

Intra-observer variability of calculated LAA orifice area, maximal and minimal diameters and depth measurements for the Rotational 3DTEE were 3.9, 2.8, 4.3 and 3.5 %, respectively, and for the CT scan method: 3.7, 2.3, 3.9 and 2.7 %, respectively, indicating a reasonable range of variability for this technique. The same observer concordance of different measurements was with good agreement (*r* = 0.95, 0.94, 0.95 and 0.97, respectively) for the Rotational 3DTEE and *r* = 0.95, 0.94, 0.99, 0.95, respectively, for the CT scan method.

## Discussion

Precise knowledge of LAA anatomy and dimensions has become a key guiding stage before implanting LAA closure devices [7, 9–13, 24]. Bedside RT3DTEE measurements of LAA maximal orifice diameter were shown to be more accurate than 2DTEE and are as accurate as CT as the gold standard [16]. However, not all echo laboratories have the capability of measuring direct online 3D images, but do have the capability of turning 3D images into 2D ones “online” for bedside measurements.

In this study we introduced a simpler and faster 3D method: it uses a rotation of the sagittal plane (green box, x-plane), that enables the operator to rapidly choose the image which gives him all the LAA parameters needed for the invasive procedure in one single “stop shop” image. This includes number of lobes, orifice area, maximal and minimal diameters and LAA depth. In contrast to the usual time consuming MPR methods, in which each of the above parameters are taken in different angles (0°, 45°, 90°, 135°) and frames, using our rotation, we could easily find the “stop shop” image point where all LAA parameters are measured (Fig. 1).

Thus, rotational 3DTEE (Yosefy rotation) may facilitate percutaneous LAA closure procedure for stroke prophylaxis in patients with non-valvular AF by choosing the appropriate LAA closure-device size. As it is simple and fast it can be repeated as many times needed before and during the procedure to ensure device size and proper implantation and LAA closure. Its accuracy is emphasized by the Bland-Altman analysis that shows good correlation and low variability between LAA orifice area, maximal and minimal diameters and depth measured by Rotational 3DTEE (3D Rotate) and by CT scanning (Fig. 6a-d). Also, the calculated differences between LAA orifice area, maximal and minimal diameters and depth were concordant (*r* = around 0.95), indicating a reasonable range of variability for this technique.

Recent evolving data show better performance of RT3DTEE for the assessment of LAA anatomy compared with 2DTEE [13, 15, 16, 20–22]. Nucifora et al. [20] have published a study showing that RT3DTEE is more accurate than 2DTEE for assessment of LAA orifice area: 137 patients (99 of them had AF) underwent 2DTEE and RT3DTEE, with CT used as a reference in 46 of them. RT3DTEE showed higher correlation with CT for the assessment of LAA orifice area, compared with 2DTEE. Our group showed in a previous study [16] that although no difference was found between LAA depth measurements using RT3DTEE and 2DTEE, compared to measurements with the direct RT3DTEE method, in 23.3 % of patients the commonly “recommended” 135° 2DTEE was not the cut plane angulation with maximal orifice diameter, thus underestimating this diameter and potentially complicating proper implantation of the device. In contrast, bedside RT3DTEE LAA measurements were not statistically different from those with CT.

Our method to size the LAA is different from the three methods that are already being used. The three methods include: the standard 2D methods using four angles (0°, 45°, 90°, 135°) [7], the 3D multiplane reconstruction (MPR) method using the three orthogonal planes from the three original pyramid 3D volume dataset [20], and the 3D TEE with on-image caliper measurement, as described in our previous work [16]. The main difference in our current technique is that we do not use different MPR orthogonal planes to find each of the LAA parameters, but we use a “stop shop” image point where all LAA parameters are measured. Thus, this technique enhances the time and simplifies LAA measurements, as shown in the attached video clip and in Fig. 1.

Thus, RT3DTEE is slowly evolving as a first-line method for sizing LAA due to its accuracy and its real-time and inherent bedside capabilities [16, 25, 26]. Compared to CT and MRI, it is faster, cheaper, and more comfortable to access; and as opposed to CT it is not associated with radiation exposure and contrast administration, while providing high quality images [27]. It may lower the cost of LAA closure procedure by also avoiding the need to exchange unsuitable devices and may reduce the risk of complications by lowering the number of failed attempts due to inappropriate device size [28, 29]. These advantages are in addition to its excellent capabilities in ruling out LA thrombus prior to the procedure [30].

The closure device is available in various sizes to accommodate individual variations on LAA anatomy. Proper positioning and sizing are essential for safety and efficacy (ref). Undersizing of the device has the potential risk of device migration or embolization and may favor peri-device leakage. Oversizing of the device should also be avoided because this may cause cardiac perforation, pericardial effusion, and cardiac tamponade.

Important aspects for LAA occlusion include the correct sizing of the landing zone diameters for the selected device and the measurement of the depth and orientation of the main anchoring lobe and the number and origin of additional lobes. Because of the substantial variations in LAA anatomy that impact device selection and efficacy, an accurate assessment of anatomic LAA characteristics is crucial before an LAA closure procedure [24].

The choice of an appropriate occlusion device depends on accurate measurements of the landing zone diameters. To achieve a secure and stable device position, the size of the occlusion device is usually selected to be a few millimeters larger in diameter than the measurements of the landing zone. The maximum length of the anchoring lobe has to be measured in addition (in the expected axis of the device) to ensure that this lobe has enough space to accommodate the selected device. Different device types require different measurements because the different occluder systems vary slightly. The angle between the ostium, the neck, and the main anchoring lobe should be evaluated because it can influence the choice of the puncture site and/or the curve of the delivery sheath. The number and origin of additional LAA lobes also needs to be assessed. Some LAA morphologies are more challenging for device closure than others; thus, LAA anatomy should be defined before any planned closure-device procedure [24].

Previous works [1, 20] used MPR for measurements on the 3D images and only as a second stage analysis do they measure LAA size by using the cut planes of each lobule at the X,Y,Z axes. Our group described a new method that measures LAA size directly on the 3D image [16]. The advantages of this method are its efficiency and speed. The measurements are given immediately, bedside, without the need for further analysis of the image.

Many echo laboratories have the capability of turning 3D images into 2D ones “online” for bedside measurements but some of them do not have the capability of measuring direct online 3D images (i.e., RT3DTEE) as we have shown before [16]. Thus, we tried to use a simpler, practical method for measuring LAA size that combines the accuracy and real time capabilities of 3D echo on one hand, and the simplicity and bedside availability of 2D echo on the other hand.

We showed that a fast and simple rotation of the introducer that demonstrates maximal diameter of LAA orifice on the 3D image (Yosefy rotation) and LAA measurements after turning the images into 2D (Rotational 3DTEE) has a similar accuracy to that of the former direct measurement on 3D image (RT3DTEE) and to CT. Our 3D protocol for LAA dimensions measurements was described in detail. We showed that rotational 3DTEE measurements of LAA were not statistically different from RT3DTEE and from CT regarding number of lobes, internal area of LAA orifice, maximal LAA diameter, and LAA depth. We believe that either RT3DTEE or Rotational 3DTEE should replace CT scan before and during implantation of percutaneous LAA occlusion devices.

### Limitations

Whenever there is a need for volume measurement by 3D echocardiography, MPR can be applied (by using cut planes of each lobule at the X,Y,Z axes, calculating the volume and adding to the volume of the other lobes). However, this method is not practical, is slow and inefficient, and thus was not used in our 3DTEE studies.

## Conclusions

Rotational 3DTEE method is a fast, simple and feasible method that has similar accuracy as RT3DTEE and CT in assessing LAA anatomy. Thus, bedside rotational 3DTEE may facilitate LAA closure procedure by choosing the appropriate device size.

## Abbreviations

“Yosefy rotation”: Rotational 3DTEE
2DTEE: 2D transesophageal echocardiography
3DTEE: 3D transesophageal echocardiography
AA: Atrial fibrillation
BSA: Body surface area
CT: Computed tomography
IVS-D: Inter-ventricular septum diameter
LAA: Left atrial appendage
LPW-D: Left posterior ventricular wall diameter
LVEDD: Left ventricular end diastolic diameter
LVEF: Left ventricular ejection fraction
LVESD: Left ventricular end systolic diameter
MPR: Multiplanar reconstruction
MRI: Magnetic resonance imaging
MS: Mitral stenosis
OAC: Oral anticoagulation
PE: Pulmonary embolism
PVI: Pulmonary vein isolation
RT3DTEE: Real-time-3D image
TEE: Transesophageal echocardiography
TTE: Transthoracic echocardiography
